# Influence of intensive lipid‐lowering on CT derived fractional flow reserve in patients with stable chest pain: Rationale and design of the FLOWPROMOTE study

**DOI:** 10.1002/clc.23895

**Published:** 2022-09-03

**Authors:** Martin B. Mortensen, Niels‐Peter Sand, Martin Busk, Jesper M. Jensen, Erik L. Grove, Damini Dey, Nadia Iraqi, Adam Updegrove, Tim Fonte, Ole N. Mathiassen, Susanne Hosbond, Hans E. Bøtker, Jonathon Leipsic, Jagat Narula, Bjarne L. Nørgaard

**Affiliations:** ^1^ Department of Cardiology Aarhus University Hospital Aarhus Denmark; ^2^ Department of Cardiology Southwestern Hospital Esbjerg Denmark; ^3^ Department of Cardiology Lillebælt Hospital Vejle‐Kolding Denmark; ^4^ Department Clinical Medicine, Faculty of Health Aarhus University Aarhus Denmark; ^5^ Biomedical Imaging Research Institute Cedars‐Sinai Medical Center Los Angeles California USA; ^6^ HeartFlow Redwood City California USA; ^7^ Division of Cardiology and Radiology St Paul's Hospital Vancouver British Columbia Canada; ^8^ Icahn School of Medicine Mount Sinai New York USA

**Keywords:** angina, coronary artery disease, fractional flow reserve, imaging, lipid lowering, statins, testing

## Abstract

**Introduction:**

Coronary CT angiography (CTA) derived fractional flow reserve (FFR_CT_) shows high diagnostic performance when compared to invasively measured FFR. Presence and extent of low attenuation plaque density have been shown to be associated with abnormal physiology by measured FFR. Moreover, it is well established that statin therapy reduces the rate of plaque progression and results in morphology alterations underlying atherosclerosis. However, the interplay between lipid lowering treatment, plaque regression, and the coronary physiology has not previously been investigated.

**Aim:**

To test whether lipid lowering therapy is associated with significant improvement in FFR_CT_, and whether there is a dose–response relationship between lipid lowering intensity, plaque regression, and coronary flow recovery.

**Methods:**

Investigator driven, prospective, multicenter, randomized study of patients with stable angina, coronary stenosis ≥50% determined by clinically indicated first‐line CTA, and FFR_CT_ ≤ 0.80 in whom coronary revascularization was deferred. Patients are randomized to standard (atorvastatin 40 mg daily) or intensive (rosuvastatin 40 mg + ezetimibe 10 mg daily) lipid lowering therapy for 18 months. Coronary CTA scans with blinded coronary plaque and FFR_CT_ analyses will be repeated after 9 and 18 months. The primary endpoint is the 18‐month difference in FFR_CT_ using (1) the FFR_CT_ value 2 cm distal to stenosis and (2) the lowest distal value in the vessel of interest. A total of 104 patients will be included in the study.

**Conclusion:**

The results of this study will provide novel insights into the interplay between lipid lowering, and the pathophysiology in coronary artery disease.

## 1 INTRODUCTION

**Figure 1:**
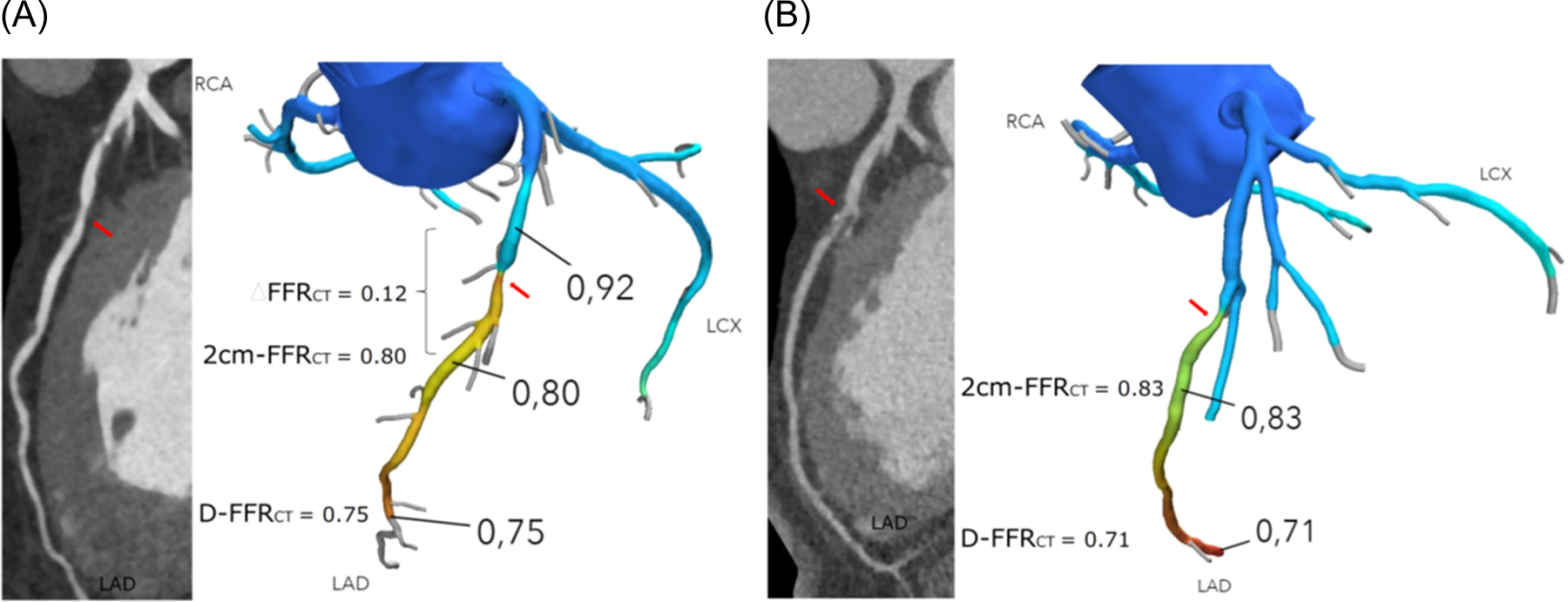
Fractional flow reserve (FFR_CT_) interpretation. (A) Lesion‐specific ischemia. There is a loss in FFR_CT_ from 0.92 to 0.80 20 mm distal to the mid‐LAD 60% diameter stenosis (red arrows). The resultant ΔFFR_CT_ is 0.12, while the lowest terminal vessel (diameter > 1.8 mm) FFR_CT_ (d‐FFR_CT_) value is 0.75. FFR_CT_, CT angiography (CTA) derived fractional flow reserve; (B) FFR_CT_ ≤ 0.80 in distal vessel segments (diameter > 1.8 mm). There is a gradual decline in downstream FFR_CT_ values with FFR_CT_ > 0.80 20 mm distal to the mid‐prox LAD stenosis (red arrows) and ≤0.80 in more distal segments with the lowest terminal vessel FFR_CT_ (d‐FFR_CT_) = 0.72. LAD, left anterior descending artery.

Fractional flow reserve (FFR) has emerged as the gold standard for assessment of lesion‐specific ischemia and decision‐making on coronary revascularization.^1^, ^2^, ^3^ It is well documented that revascularization can be safely avoided in lesions with FFR > 0.80, while patients having one or more lesions with FFR ≤ 0.80 may benefit from revascularization.^1^, ^4^ In the FAME‐2 study of patients with coronary artery disease (CAD) and FFR ≤ 0.80, the incidence of the composite endpoint (death, myocardial infarction, and revascularization) was lower in the revascularization than in the medical therapy group (13.9 vs. 27%).^4^ Notably, the driving force for the difference in outcomes was repeat revascularization, while the majority of patients in the medically treated group did not experience any serious cardiac events at 5‐years follow‐up (all‐cause death or myocardial infarction did not occur in 85.9% of the patients).^4^ Coronary CT angiography (CTA) derived FFR (FFR_CT_) is based on standard acquired CT data set postprocessing algorithms with integration of quantitative anatomical, physiological modeling and computational fluid dynamics.^5^ In patients with stable CAD, FFR_CT_ demonstrates high diagnostic performance relative to invasively measured FFR, and in real‐world practice FFR_CT_ may favorably change patient management and outcomes.^6^, ^7^, ^8^, ^9^, ^10^, ^11^, ^12^ Accordingly, in the recent 2021AHA/ACC/ASE/CHEST/SAEM/SCCT/SCMR Guideline for the evaluation and diagnosis of chest pain, it is stated that FFR_CT_ in intermediate‐high risk patients with chest pain and stenosis 40%–90% (mid‐proximal segment on CTA) can be useful for the diagnosis of vessel‐specific ischemia and to guide clinical decision‐making on revascularization (evidence level, 2A).^13^ A negative FFR_CT_ result (>0.80) in patients with intermediate range coronary lesions is associated with favorable clinical outcomes.^10^, ^11^, ^12^ A positive FFR_CT_ result (≤0.80) indicates lesion‐specific ischemia when positive 1–2 cm distal to a stenosis (similar to FFR) or diffuse ischemia when there is no lesion‐specific ischemia but a gradual decline in FFR_CT_ along the length of one or more vessels with distal values ≤0.80 (Figure 1).^10^, ^14^ While patients with lesion‐specific ischemia by FFR_CT_ may be referred to ICA for possible revascularization, patients without a focal FFR_CT_ loss have no focal substrate for coronary intervention, and thus are managed by optimal medical therapy alone.^14^

Lipid lowering therapy with statins is the cornerstone of contemporary preventive care in patients with CAD. Statin therapy is associated with plaque stabilization through favorable changes in high‐risk atherosclerotic phenotype characteristics (APC) and a lower rate of overall atherosclerotic plaque volume progression.^15^, ^16^, ^17^, ^18^ These changes include a reduction in the total noncalcified plaque burden including low attenuation density plaques (LAP), and stabilization of thin‐cap fibroatheroma. By serial coronary CTA, a significant decrease in APC plaque volumes have been demonstrated over 6–12 months both by regular and potent statin therapy, with more pronounced effects of the latter.^17^ In post‐hoc analyses, the presence and burden of APC plaques, even in nonobstructive CAD, is associated to the presence and severity of ischemia as assessed by FFR.^19^, ^20^, ^21^ One prospective single‐center study of 20 patients with stable CAD treated with fixed‐dose rosuvastatin 5 mg per day for 18 months after coronary stenting suggested the existence of a negative correlation between low density lipoprotein (LDL) cholesterol lowering and changes in FFR.^22^ We designed the prospective, multicenter, randomized FLOWPROMOTE study to investigate, in hemodynamically significant CAD, whether lipid lowering‐induced changes in plaque morphology improves coronary physiology as assessed by FFR_CT_.

## 2 METHODS

### 2.1 Study design

The FLOWPROMOTE study is an investigator‐driven, proof‐of‐concept, prospective, multicenter, randomized, open‐label trial. The study is registered at www.ClinicalTrials.gov (NCT04737408). Three Danish centers with experience in coronary CTA and FFR_CT_ testing participate in the study (Department Cardiology, Aarhus University Hospital; Department of Cardiology, University Hospital of Southern Lillebælt Hospital; and Department of Cardiology, University Hospital of Southern Denmark, Esbjerg, Denmark). A total of 104 patients will be included in the study. The study protocol has been approved by the Danish Medicines Agency, the Ethics Committee (EudraCT nr. 2019‐001912‐50) and by the research review boards at each participating center. The study is led by a chairman, primary investigator, principal investigators and a steering committee (details are provided in the Supporting Information). Coronary plaque and FFR_CT_ analyses will be analyzed by independent core‐laboratories. Details of the committees, laboratories including members are provided in the Supporting Information.

### 2.2 Study objectives

The main purpose of study is two‐fold: (1) to assess whether lipid lowering therapy in stable CAD patients with FFR_CT_ ≤ 0.80 is associated with improvement in coronary physiology, and (2) to investigate whether there is a dose–response relationship between lipid lowering intensity, plaque regression, and coronary flow recovery.

### 2.3 Study subjects

**Figure 2:**
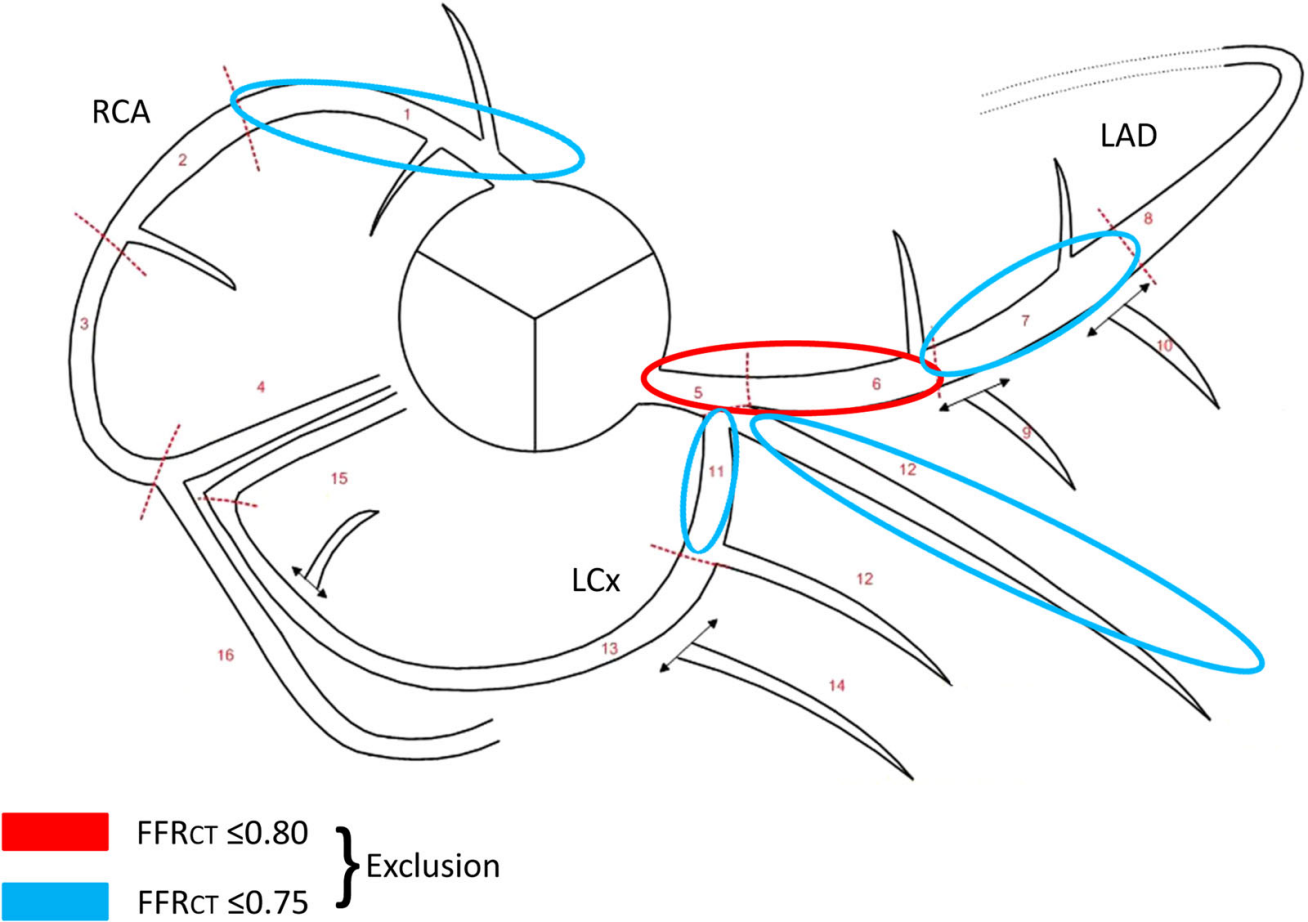
Fractional flow reserve (FFR_CT_) based exclusion criteria. Patients with FFR_CT_ ≤ 0.80 in left main stem, or proximal LAD, and those with FFR_CT_ ≤ 0.75 in a dominant proximal RCA, dominant proximal LCx, ramus or mid‐LAD segment are excluded from this study. LCx, left circumflex artery; RCA, right coronary artery.

Patients referred for nonemergent clinically indicated coronary CTA demonstrating CAD, diameter stenosis ≥50%, FFR_CT_ ≤ 0.80, and no obvious indication for coronary revascularization are eligible for study inclusion (Table 1). In the participating institutions, FFR_CT_ testing is recommended for physiological coronary assessment in patients with stable chest pain and intermediate‐risk anatomy as previously described.^10^ Anatomical and physiological study eligibility criteria are shown in Table 1 and Figure 2. All patients undergoing clinically indicated FFR_CT_ assessment (including reasons for study exclusion) during the study period are registered in a screening list.

**Table 1:** Study eligibility criteria

Inclusion criteria	Exclusion criteria
1. Age >35 years 2. Symptoms suggestive of stable CAD 3. CAD with at least one stenosis with ≥50% lumen reduction determined by the index coronary CTA investigation^a^ 4. Presence of at least two low attenuation plaques (with attenuation <30 Hounsfield units) present in at least two orthogonal planes by CTA 5. Sinus rhythm 6. LDL cholesterol >2.0 mM 7. FFR_CT_ ≤ 0.80 (Figure 2) 8. Life expectancy >3 years 9. Fertile women must use safe contraception throughout the study period	1. Previous lipid lowering therapy^b^ 2. Known CAD 3. Unstable angina 4. Indication for coronary revascularization 5. BMI > 40 6. Allergy to iodinated contrast media 7. Poor coronary CTA image quality inadequate for FFR_CT_ calculation (determined by core laboratory) 8. Pregnancy (women < 45 years will be screened for pregnancy) 9. Moderate to severe liver failure 10. Estimated glomerular filtration rate <60 ml/min 11. Participation in another clinical trial **Anatomical or FFR**_**CT**_ **based exclusion criteria**: 12. Left main‐ stenosis ≥50%, 3‐VD or high‐grade proximal LAD stenosis resulting in direct referral to ICA 13. FFRct ≤ 0.80 2 cm distal to stenosis on CTA in segments 5 and 6 (Figure 2). 14. FFRct ≤ 0.75 2 cm distal to stenosis on CTA in segments: 1^c^, 7, 11^c^, 12 (ramus) (Figure 2).

Abbreviations: BMI, body mass index; CAD, coronary artery disease; CTA, computed tomography angiography; FFRCT, CTA derived fractional flow reserve; ICA, invasive coronary angiography; LAD, left anterior descending coronary artery; LDL, low density lipoprotein.

^a^ Assessed at the discretion of the CTA reading cardiologist.

^b^ Patients treated with lipid lowering therapy <3 months before the index CTA investigation can be included in the study if meeting inclusion criteria.

^c^ If (co‐) dominant vessel.

### 2.4 Patient workflow

**Figure 3:**
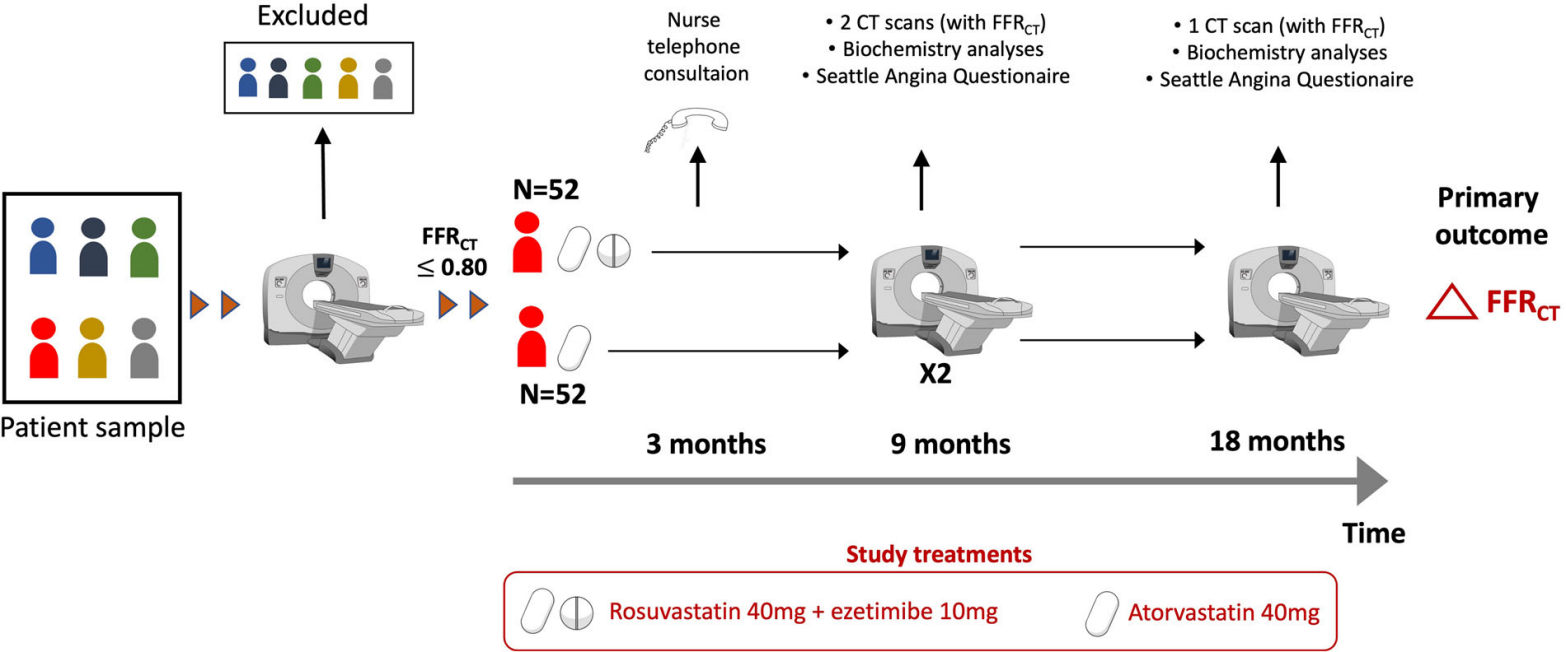
Study flow. At the 9 months visit two separate CT angiography scans are performed with a 1 h interval.

Patients randomized to one of two lipid lowering treatment regimens are followed for 18 months (Figure 3). Coronary CTA scans with blinded plaque and FFR_CT_ analyses will be repeated after 9 and 18 months. For assessment of the reproducibility of plaque and FFR_CT_ two CTA scans at a 1 h interval are performed at the 9 months visit. Antianginal and antiplatelet treatments are used at the discretion of the treating physicians. Recurrent angina will be managed according to societal guidelines, including referral to ICA in the event of uncontrolled symptoms.^2^, ^3^, ^13^ In the event of revascularization being performed during follow‐up, patients are encouraged to continue in the study (including the CT imaging protocol for plaque and FFR_CT_ assessments in nonrevascularized vessels).

### 2.5 Coronary CT angiography

CTA is performed using contemporary high‐end technology scanners according to the best practice CTA acquisition guidelines.^23^ Oral and/or intravenous beta‐blockers or oral ivabradine are administered if necessary targeting a heart rate <60 beats/min., and all patients receive sublingual nitrates (spray, 0.8 mg) 3 min before the scan. An initial nonenhanced scan for assessment of the Agatston score is performed. Coronary CTA acquisition using prospective electrocardiographic triggering with 70–140 kV tube voltage depending on patient weight and the calcific burden is recommended. CTA acquisition settings in the index scan are registered for repetition in the follow‐up CTA investigations. Coronary stenosis severity at the index scan is assessed at the discretion of the CTA reading cardiologists. Lesion location is registered in an 18 segment coronary model.^24^ Patients with at least one lesion with ≥50% stenosis severity at the index scan will have FFR_CT_ performed.

### 2.6 Biochemistry

Measurement of total cholesterol, high density lipoprotein cholesterol, LDL cholesterol is measured at baseline and at follow‐up visits. Lipoprotein(a) is measured at the baseline visit. Hepatic enzymes, creatin kinase, creatinine, and hemoglobin is assessed at baseline, and after 3 months. Further special analyses related to inflammation for substudy analyses will be performed (Supporting Information).

### 2.7 Coronary plaque analysis

Together with the pericoronary adipose tissue (PCAT) attenuation as a marker of coronary artery inflammation, plaque analyses will be performed at a core‐lab in vessel segments with diameter ≥2 mm using a semiautomated software (Autoplaque, Cedars‐Sinai Medical Center) as previously described.^18^, ^19^, ^20^, ^25^, ^26^, ^27^, ^28^ In brief, automated attenuation thresholds will be used for scan‐specific plaque differentiation,^26^, ^27^ and the vessel lumen, wall and plaque are defined automatically with manual input as required. Total plaque, calcified, and noncalcified (including LAP < 30 HU) will be measured (mm^3^), and aggregate plaque volume (APV%) will be computed as total plaque volume/vessel volume × 100%. Plaque burden will be assessed on a per‐lesion (diameter stenosis ≥50%), vessel and patient level. Observers will be blinded to all clinical information, timing of the CT scans and FFR_CT_ results.

### 2.8 CTA derived fractional flow reserve

**Figure 4:**
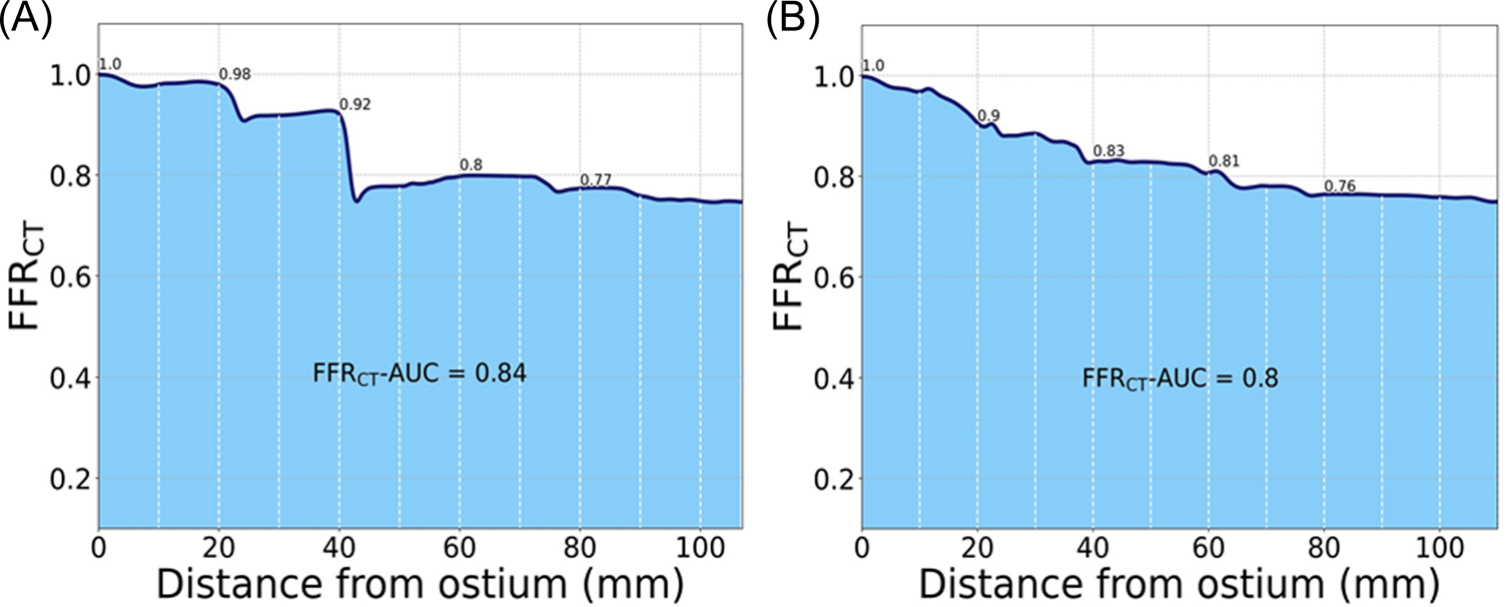
“Total vessel” FFR_CT_, FFR_CT_‐AUC. All FFR_CT_ values at 1 mm intervals from the ostium along the total length of the vessel (including segments with a diameter >1.8 mm) are integrated by using the normalized AUC (FFR_CT_‐AUC) index and hereby assessing the “total vessel” FFR_CT_. The FFR_CT_‐AUC index thus represents the sum of all multiple downstream resistances from both focal and diffuse disease. We hypothesize that LDL lowering will lead to an upward shift of the FFR_CT_ curve and thus an increase in FFR_CT_‐AUC in vessels with CAD. (A) is calculated from case A, and (B) from case B in Figure 1. AUC, area under the curve; CAD, coronary artery disease.

The FFR_CT_ computation data transferal process has previously been described in detail.^5^, ^6^, ^7^ FFR_CT_ computations will be performed by technicians without knowledge of patient characteristics or timing of the CT scans (HeartFlow Inc). FFR_CT_ ≤ 0.80 is considered indicative of the presence of ischemia. The following FFR_CT_ variables will be registered for comparisons: (1) FFR_CT_ 2 cm's distal to the stenosis, (2) the translesional FFR_CT_ gradient (ΔFFR_CT_ = value 1 cm proximal to the upper border of the stenotic plaque ÷ FFR_CT_ 2 cm distal to the plaque, Figure 1), (3) the lowest terminal vessel (diameter > 1.8 mm) FFR_CT_ value, (4) the number of vessels with ΔFFR_CT_ ≥ 0.06,^10^, ^14^, ^29^ and (5) a new “total vessel” FFR_CT_ index (FFR_CT_–AUC) will be assessed for temporal comparisons (Figure 4). The FFR_CT_–AUC integrates all values by using the normalized area under the curve index. FFR_CT_–AUC, lesion‐specific (when applicable) and terminal vessel FFR_CT_ values will be assessed in all vessels, including those with diffuse CAD without stenosis and with FFR_CT_ > 0.80. Lesions and segments of interests will be identified on a blank 3‐dimensional computer model, followed by blinded integration of the relevant FFR_CT_ values ensuring comparison of corresponding FFR_CT_ data. For sub‐study analysis the coronary volume‐to‐myocardial mass (V/M) and the ischemic myocardial burden as assessed by the APPROACH_FFRCT_ score will be calculated.^30^, ^31^

### 2.9 Randomization and treatment

Patients are randomized 1:1 to either usual care lipid lowering treatment (atorvastatin 40 mg daily) or to intensive lipid treatment (rosuvastatin 40 mg plus ezetimibe 10 mg daily) for 18 months. We expect that atorvastatin 40 mg will reduce LDL cholesterol with 45%–50% while the combination of rosuvastatin 40 mg + ezetimibe 10 mg is expected to reduce LDL cholesterol with up to 70%.^32^ Study medication is provided to each patient in 3‐month batches and unused medication is returned for assessment of medication adherence. New or residual angina or potential statin side effects are addressed at the 3‐month visits or via a direct telephone line to the research staff in between these visits. Thus, we expect a low rate of study medication nonadherence and low loss of follow‐up. In the event of statin side‐effects, the rosuvastatin dose may be lowered to 20 mg in the intensive treatment arm. If continuing intolerable side effects patients will be withdrawn from their study allocated treatment to no or alternative downstream lipid lowering management according to local practice (observation group). In Denmark, the patients who fulfill the FLOWPROMOTE inclusion criteria have an LDL‐cholesterol treatment goal of <1.8 mM and are recommended treatment with atorvastatin 40–80 mg or rosuvastatin 20 (−40) mg. As atorvastatin 40 mg reduces LDL‐cholesterol by 45%–50%,^32^ we estimate that most individuals in the atorvastatin 40 mg group will reach an LDL‐cholesterol level in the range of 1.5–2.0 mM. In the event that LDL‐cholesterol is considered to be uncontrolled by the treating physician, patients may have their lipid‐lowering treatment intensified. Patients with changes in the assigned study medication during the course of the study will be followed in the observation group. Except for the study directed treatment regimen, the observation group will follow the study protocol according to the intention‐to‐treat principle including CTA studies with blinded plaque and FFR_CT_ analyses.

### 2.10 Endpoints and substudy analyses

In our analyses, we will assess whether the changes in FFR_CT_ are associated to the absolute changes in LDL‐cholesterol from baseline until 18 months of follow‐up (ΔLDL‐cholesterol). The primary endpoint will be the 18‐month difference in FFR_CT_ using (1) the FFR_CT_ value 2 cm distal to stenosis in the event of lesion‐specific ischemia at baseline, and (2) the lowest distal value in patients with a gradual nonfocal downstream decline in FFR_CT_ (Figure 1). Secondary endpoints are the temporal difference in ΔFFR_CT_ and FFR_CT_‐AUC, and changes in FFR_CT_ between the two treatment groups. Additional exploratory analyses are outlined in the Supporting Information. Substudies are planned to assess (1) the interscan reproducibility (based on the two CT scans at 9 months, Figure 3) of total and individual plaque volumes, and PCAT attenuation estimates, FFR_CT_, FFR_CT_‐AUC, ΔFFR_CT_, APPROACH_FFRCT_ score and the V/M‐ratio, and (2) the relationship between ΔLDL‐cholesterol, total and LAP volumes, and changes in inflammatory biomarkers, and FFR_CT_ estimates.

### 2.11 Sample size

We assume that 104 patients will be sufficient to demonstrate a dose‐relationship effect between LDL lowering and FFR_CT_ recovery. Assuming an average temporal average increase in the lowest FFR_CT_ distal value of +0.07 in the intensive lipid treatment group, and +0.04 in usual care lipid lowering group (personal experience following treatment with atorvastatin 40–80 mg over 5–9 months, *n* = 9), with a noncompliance rate of 15% in both groups, leaving an intensity to treat effect of 0.85 × (0.07 ÷ 0.04) = 0.025, and a standard deviation of the difference = 0.03 in both groups, one can with 99% power detect a statistical difference between the randomization groups (and with 72% power to detect an effect >0.01).

### 2.12 Data analysis plan

In the primary intention‐to‐treat analysis plan changes in FFR_CT_ will be compared in a temporal hierarchically fashion. Thus, if the difference in FFR_CT_ estimates at 18 months is of statistically significance, then the difference in FFR_CT_ at 9 months (scan 1) will also be tested. Changes in categorical variables will be analyzed using the Fishers exact test, and means between groups by the Student *t*‐test, Wilcoxon signed rank test or the Kruskal–Wallis test as appropriate. The continuous relationship between variables will be assessed by linear regression supplemented by restricted cubic spline to adjust for nonlinearity as appropriate. Reproducibility assessments will be performed by calculation of standard error of measurement and within‐subject coefficient of variation.

### 2.13 Ethical considerations and safety

This study is conducted in accordance with the Declaration of Helsinki, thus written informed consent will be obtained from each participant at study inclusion. The study is approved by the Ethics Committee for each participating center. Regulations for good clinical practice (GCP) will be followed and monitored by the GCP‐units at Aarhus University and University of Southern Denmark Hospitals. All patients in the FLOWPROMOTE study will have a strong guideline‐recommendation for initiating lipid‐lowering therapy with statins and/or ezetimibe.^3^, ^13^ Statins and ezetimibe, even in combination, have proven safe.^33^ The radiation exposure for each CT scan will be approximately 2.5–3.0 mSv, thus in total 10–12.5 mSv for patients participating in the study corresponding to or less than the radiation exposure inflicted by a single rest/stress single‐photon emission computed tomography (SPECT).^34^ Deferring patients with lesion‐specific ischemia from ICA and revascularization will involve only patients with controlled symptoms and without proximal hemodynamically significant lesions in whom revascularization does not improve clinical hard outcomes when compared to medical treatment alone.^35^, ^36^, ^37^ Inherently, we do not expect revascularization performed during the study period to significantly influence study results. Overall, the FLOWPROMOTE study is unlikely to cause serious side effects and inclusion in the study does not possess any known pharmacological harm or management disadvantage to study patients beyond what is expected in routine clinical practice.

### 2.14 Study status

Inclusion of patients began May 2020. Inclusion has been temporary halted between December 2020 until April 2021 due to the Covid lock‐down. Currently 97 patients have been included in the study. Patient enrollment completion is expected by November 2022.

## 3 DISCUSSION

The concept of identifying and revascularizing obstructive coronary plaques with the intent to relieve symptoms and improve long‐term outcome is receiving much attention in contemporary clinical practice. Accordingly, an FFR‐guided revascularization strategy is recommended by European and American guidelines to improve the identification of ischemia producing stenoses that can be targeted by percutaneous coronary intervention (PCI) while deferring revascularization in patients with nonischemic lesions (i.e., FFR > 0.80).^1^, ^2^, ^3^ The conceptual basis behind this prevailing strategy is the general assumption that reducing myocardial ischemia improves clinical outcomes. However, results from recent clinical trials such as FAME‐II and ISCHEMIA have questioned this long‐held belief as they have failed to demonstrate risk reduction of myocardial infarction or death with PCI versus contemporary optimal medical therapy.^4^, ^37^ Thus, the clinical value of routinely revascularizing all stenoses with FFR ≤ 0.80 is limited since only a relatively small fraction of FFR positive lesions cause adverse events if these lesions were left to medical treatment alone.^4^ Further, a less discussed but equally important question is whether a given FFR value in the coronary artery is fixed or whether it can be improved by pharmacological management. Given the many beneficial changes in plaque phenotype characteristics that are observed with lipid lowering therapies, including reduction in LAP volumes^15^, ^16^, ^17^, ^18^, ^38^, ^39^, ^40^ and recent data demonstrating that such features are independent predictors of lesion‐specific ischemia expressed by abnormal FFR^19^, ^20^, ^21^ we speculate that such treatment may also improve the coronary physiology, that is, by increasing FFR values in segments with obstructive atherosclerosis. It is on the basis of this hypothesis that the FLOWPROMOTE study was initiated.

FFR is invasive and associated with increased risk. Therefore, in this study employing repeated assessments of the coronary physiology, FFR_CT_ is used as a surrogate for FFR. This strategy is supported by several studies demonstrating high diagnostic performance and correlation when compared to measured FFR.^6^, ^7^, ^8^ FFR_CT_ provides simultaneous computation of pressure and flow in the entire coronary tree, thus exposing both lesion‐specific pressure as well as nadir FFR_CT_ values. Low terminal vessel FFR_CT_ values remote from a focal lesion may be due to diffuse CAD and/or reflect the sum of serial flow‐limiting lesions.^14^ In a previous study of real‐world consecutive patients with stable CAD, approximately 50% of those with FFR_CT_ values ≤0.80 deferred from catheterization had low terminal vessel FFR_CT_ positivity.^10^ We hypothesize that both lesion‐specific as well as low terminal vessel FFR_CT_ values ≤0.80 can be increased by lipid lowering therapy. On the other hand, lipid lowering‐induced plaque regression/stabilization with associated improvements in the physiological profile expectedly is not restricted to lesions or vessels with FFR_CT_ ≤ 0.80. To delineate in more detail the total effect of plaque regression on the coronary physiology, we introduce in this study the FFR_CT_‐AUC index which integrates all downstream resistances arising from both focal and diffuse disease. When compared to the terminal vessel FFR_CT_ (also addressing the sum of focal and diffuse disease), FFR_CT_‐AUC may be less susceptible to the potential influence of the small vessel caliber and suboptimal CT resolution on the FFR_CT_ diagnostic accuracy. Thus, relative to traditional FFR_CT_ metrics, FFR_CT_‐AUC may add important information on the impact of lipid lowering therapy on flow both in vessels with or without FFR_CT_ ≤ 0.80 at baseline. The repeated CTA + FFR_CT_ investigation strategy in the FLOWPROMOTE study allows for comprehensive assessment of the temporal interplay between lipid lowering therapy, changes in disease morphology and physiology including new metrics such as PCAT attenuation, V/M ratio and the APPROACH_FFRCT_ score.^25^, ^30^, ^31^

In the FLOWPROMOTE study, patients are randomized to two different lipid lowering regimens; usual care with atorvastatin 40 mg/day versus intensive lipid lowering with rosuvastatin 40 mg/day plus ezetimibe 10 mg/day. A no‐treatment arm was not included because of the obvious unethical nature of omitting guideline‐directed lipid lowering treatment in patients with documented CAD. The rationale for choosing the two different treatment intensities is to be able to demonstrate a potential dose–response relationship between achieved LDL cholesterol values and improvements in coronary physiology. Such a dose–response relationship would be consistent with previous studies demonstrating that the extent of plaque volume regression is greater in patients who achieve the lowest LDL cholesterol values.^38^, ^39^ Importantly, these previous studies have not indicated that there is a lower LDL threshold beyond which further LDL lowering would not yield additional plaque regression. Accordingly, patients treated with multiple lipid‐lowering agents such as combination therapy of statin and ezetimibe or statin plus PCSK9 inhibition have greater plaque regression than patients treated with statin monotherapy.^40^, ^41^ The FLOWPROMOTE study may therefore be able to provide answers on the potential of imaging to guide personalized lipid lowering strategies based on both the atherosclerosis and physiology phenotype.

Demonstration of a beneficial influence of pharmacological lipid lowering on both the coronary plaque phenotype and concomitant improvements in coronary physiology will expand our understanding of CAD, underscore the importance of lipid lowering in patients with CAD, and potentially in the future have implications for individualized management of patients with lesion‐specific ischemia who may benefit from intensive lipid lowering therapy as an alternative to current interventional practice. If proof‐of‐concept can be demonstrated the present study may form the basis for initiation of large‐scale, randomized trials to assess the safety of deferral ICA and revascularization in subsets of patients with CTA determined flow‐obstructive CAD.

## CONFLICTS OF INTEREST

Adam Updegrove is an employee and shareholder in HeartFlow. Bjarne L. Nørgaard has received unrestricted research grants from HeartFlow and the Novo Nordic Research Foundation. Erik L. Grove has no conflicts of relevance to this manuscript. He has received speaker honoraria or consultancy fees from Astra Zeneca, Alexion Pharma, Bayer, Boehringer Ingelheim, Bristol‐Meyers Squibb, Pfizer, MSD, MundiPharma, Organon, Portola Pharmaceuticals, and Lundbeck Pharma. Erik L. Grove is an investigator in studies sponsored by Astra Zeneca and has received unrestricted research grants from Boehringer Ingelheim. Hans E. Bøtker has received unrestricted research grants from HeartFlow, and is on the Advisory board for Boehringer Ingelheim. JonathonLeipsic has received an unrestricted research grant from GE Healthcare, honoraria from Philips, consulting fees from Circle CVI and HeartFlow, and holds stock options in HeartFlow. TimFonte is an employee and shareholder in HeartFlow.

## Supporting information

Supplementary information.

## Data Availability

Data sharing not applicable—no new data generated.
