# Supplementary material for: Influence of intensive lipid‐lowering on CT derived fractional flow reserve in patients with stable chest pain: Rationale and design of the FLOWPROMOTE study
Source: Clin Cardiol. 2022 Sep 3;45(10):986–94. doi: 10.1002/clc.23895 (PMC9574753; doi:10.1002/clc.23895)
Supplement: Supplementary file 1 — Supplementary information. [file CLC-45-986-s001.docx]

**Supplemental Appendix**

**I. Participating centres, committees and laboratories**

**The participating centres and principal investigators:**

● Aarhus University Hospital, Department Cardiology, Palle Juul-Jensens Boulevard 99, 8200 Aarhus N (sponsor and coordinating center)

Bjarne Linde Nørgaard, Professor, MD, DMSc, PhD, Primary investigator (Legal representative authorized by the sponsor)

Martin Bødtker Mortensen, M.D, Ph.D. Coordinating investigator

Jesper Møller Jensen, M.D, Ph.D., Co-principal investigator

Nadia Iraqi, MD, Co-principal investigator

Erik Lerkevang Grove, Ass. Professor, MD, PhD, Co-principal investigator

● South Western Hospital, -Esbjerg, Department of Cardiology

Niels-Peter Rønnow Sand, Professor, MD, PhD, Principal investigator

● Lille Baelt Hospital, Vejle, Department of Cardiology

Martin Busk MD, PhD, Principal investigator

**Steering committee:**

1. Hans Erik Bøtker (Chairman)

2. Bjarne Linde Nørgaard (Primary investigator)

3. Martin Bødtker Mortensen (Coordinating investigator)

4. Niels-Peter Rønnow Sand (Principal investigator)

5. Martin Busk (Principal investigator)

6. Jonathon Leipsic

7. Jagat Narula

9. Damini Dey

**Statistical Analysis Core Lab:**

Professor Erik Parner, PhD

Division Biostatistics, Institute of Public Health, Aarhus University, Aarhus,

Denmark

**Coronary CTA Core Lab:**

Professor Jonathon Leipsic, MD, and Amir Ahmadi, MD.

Division of Cardiology and Radiology, University of British Columbia, St Paul’s Hospital, Vancouver, BC

Canada

**Biochemistry Core lab:** Erik Lerkevang Grove, and Anne-Mette Hvas. Department of Clinical Biochemistry, Aarhus University Hospital, Aarhus, Denmark

**Coronary Plaque Analysis Core Lab:**

Damini Dey, Ass. Professor, Ph.D.

Biomedical Imaging Research Institute, Cedars-Sinai Medical Center, LA,

USA

**FFR_CT_ analyses Core Lab:**

HeartFlow, Redwood City, Cal, US

Charles Taylor, PhD, Adam Updegrove, PhD and Tim Fonte, PhD

**Monitoring of the study according to GCP guidelines**

GCP-unit, Aalborg and Aarhus University Hospitals, Regionshuset Aarhus, Denmark

**II, FLOWPROMOTE substudy “Antiinflammatory effects of intensive lipid-lowering with atorvastatin or rosuvastatin/ezetimibe in patients with chronic coronary syndromes”** Background: Lipid-lowering is associated with a substantially reduced risk of cardiovascular events, and several studies have aimed to explore the mechanisms explaining the cardiovascular benefit of lipid-lowering. The majority of studies have focused on different aspects of coronary atherosclerosis, but there is also substantial interest in potential antiinflammatory effects that e.g. may inhibit development of vulnerable plaques and thus reduce the risk of cardiovascular events. However, the vast majority of studies exploring potential inflammatory effects of lipid-lowering drugs only measured selected inflammatory markers, such as high-sensitivity C-reactive protein. In this substudy, we will use a novel proximity extension assay to evaluate a total of 92 inflammatory biomarkers in stable coronary artery disease patients before and 18 months after initiation of lipid-lowering treatment.

Aim: To evaluate the antiinflammatory effects of 18 months lipid-lowering treatment by atorvastatin or rosuvastatin and ezetimibe.

Hypotheses: 1) Lipid-lowering treatment will reduce the level of inflammatory biomarkers, 2) Intensive lipid-lowering with once daily rosuvastatin 40 mg and ezetimibe 10 mg will have a stronger inflammatory effect than standard lipid-lowering with once daily atorvastatin 40 mg.

Patients: All patients included in the main FLOWPROMOTE study at Aarhus University Hospital will also be included in this substudy.

Biochemistry: *Collection & handling*: Blood samples are collected at baseline and after 18 months of treatment. A volume of 1,8 mL will be collected from an antecubital vein into tubes contining 5.4 mg dipotassium-ethylene-diamine-tetraacetate (Becton Dickinson). Within one hour after blood sampling, platelet-poor plasma is obtained after centrifugation for 10 minutes at 1500 *g*. Plasma samples are subsequently stored at −80 °C in two tightly capped 500 µL cryotubes (Sarstedt, VWR-Bie & Berntsen, Søborg, Denmark) at the Department of Clinical Biochemistry, Aarhus University Hospital, Aarhus, Denmark.

*Analyses*: Samples are thawed at 37 °C and analysed within one hour. Biochemical analyses are performed as a batch analysis by personnel without knowledge on study patients (after 18 months follow-up of the last included patient). An inflammation biomarker panel based on a novel proximity extension assay technique (Olink^®^ Bioscience, Uppsala, Sweden) will be used to analyse samples for 92 inflammation-related proteins as listed by the manufacturer ([www.olink.com/products/inflammation/](http://www.olink.com/products/inflammation/)). Analyses will be performed at BioXpedia A/S, Aarhus, Denmark.

**III. Secondary exploratory study assessments**

| 1. Baseline to 18-month difference in FFR_CT_-AUC for the vessel of interest* and separately for the other two vessels (Figure 3) |
| --- |
| 2. Baseline to 18-month difference in ΔFFR_CT_^†^ for the vessel of interest* and separately for the other two vessels (Figure 3) |
| 2. Baseline to 18-month difference in coronary plaque (total, LAP, non-calcified and calcified plaque) volumes, and plaque remodeling index |
| 3. Baseline to 18-month difference in perivascular fat attenuation index |
| 4. Baseline to 18-month difference in V/M ratio |
| 5. Baseline to 18-month difference in APPROACH_FFRCT_ score |
| 4. Continuous relationship between the baseline to 18-month difference in LDL, total and LAP volumes, perivascular fat attenuation index, versus differences in FFR_CT_, FFR_CT_-AUC, ΔFFR_CT_^†^ and V/M ratio and APPROACH_FFRCT_ score |
| 5. LDL-FFR_CT_ relationship according to age, gender, the presence of diabetes, smoking status, Agatston score (>0, >400), angina status, the ischemic vessel territory (LAD versus non-LAD territory), and to the pattern of ischemia (lesion-specific or diffuse non-focal CAD ischemia) |
| 6. Baseline to 18-month difference in angina severity (7-item Seattle Angina scale) |
| 7. Baseline to 18-month difference in quality of life score (5-item EuroQOL scale) |
| 8. Major cardiac events (all-cause death, myocardial infarction, unplanned revascularization or ICA) during follow-up will be registered |

*Main vessel (excluding side-branches) with lesion-specific ischemia or in the absence of lesion-specific ischemia the vessel with the lowest distal FFR_CT_ value. ^†^Translesional FFR_CT_. The APPROACH_FFRCT_ score is a CTA-FFR_CT_ derived surrogate of the ischemic myocardial burden; CTA, computed tomography angiography; FFR_CT_, CTA derived fractional flow reserve; FFR_CT_-AUC, the “total vessel” FFR_CT_ integrating all values by using the normalized area under the curve index in main vessels (excluding side branches); ICA, invasive coronary angiography; LAD, left anterior descending coronary artery; LDL, low density lipoprotein cholesterol; LAP, low attenuation density plaque; QOL, quality of life
